# Do individuals with and without depression value depression differently? And if so, why?

**DOI:** 10.1007/s11136-015-1018-3

**Published:** 2015-06-03

**Authors:** Katerina Papageorgiou, Karin M. Vermeulen, Maya J. Schroevers, Anne M. Stiggelbout, Erik Buskens, Paul F. M. Krabbe, Edwin van den Heuvel, Adelita V. Ranchor

**Affiliations:** Health Psychology Section, University Medical Center Groningen, University of Groningen, Groningen, The Netherlands; Department of Epidemiology, University Medical Center Groningen, University of Groningen, Groningen, The Netherlands; Department of Medical Decision Making, Leiden University Medical Center, Leiden, The Netherlands

**Keywords:** Depression, Discrepancies, Valuations, Health-related quality of life, Time-trade-off

## Abstract

**Purpose:**

Health state valuations, used to evaluate the effectiveness of healthcare interventions, can be obtained either by the patients or by the general population. The general population seems to value somatic conditions more negatively than patients, but little is known about valuations of psychological conditions. This study examined whether individuals with and without depression differ in their valuations of depression and whether perceptions regarding depression (empathy, perceived susceptibility, stigma, illness perceptions) and individual characteristics (mastery, self-compassion, dysfunctional attitudes) bias valuations of either individuals with or without depression.

**Methods:**

In an online study, a general population sample used a time-trade-off task to value 30 vignettes describing depression states (four per participant) and completed questionnaires on perceptions regarding depression and individual characteristics. Participants were assigned to depression groups (with or without depression), based on the PHQ-9. A generalized linear mixed model was used to assess discrepancies in valuations and identify their determinants.

**Results:**

The sample (*N* = 1268) was representative of the Dutch population on age, gender, education and residence. We found that for mild depression states, individuals with depression (*N* = 200) valued depression more negatively than individuals without depression (*N* = 1068) (*p* = .007). Variables related to perceptions of depression and individual characteristics were not found to affect valuations of either individuals with or individuals without depression.

**Conclusion:**

Since the general population values depression less negatively, using their perspective might result in less effectiveness for interventions for mild depression. Perceptions of depression or to individual characteristics did not seem to differentially affect valuations made by either individuals with or without depression.

## Introduction

Depression is a prevalent condition [1] that imposes a high burden on health-related quality of life [2–4]. Various types of interventions have been found effective in alleviating depression [5–7]. Economic evaluations of healthcare interventions are currently necessary for policy decisions. Cost-utility analysis is a type of economic evaluation in which health state valuations (also called “utility values,” “utilities” or “preferences”) are the recommended metric measures to assess the effectiveness of the intervention [8]. Currently, cost-utility analyses are primarily used in the evaluation of interventions for somatic conditions but are less frequently used in evaluating interventions for mental conditions such as depression [9].

A question that has generated ongoing discussion, with possibly serious policy implications, is whose values should be used in evaluating health conditions: those expressed by the patients experiencing the health states or those expressed by the general population imagining them [10]. This issue has been extensively debated [11] in light of evidence showing that patients and the general population differ in their valuations of health conditions. Theoretically, the general population should be less biased in their valuations due to lack of self-interest; on the other hand, patients should be better informed on their conditions. Studies on valuations of somatic conditions show that somatically ill patients tend to value their states less negatively than the general population [12, 13], although this finding is not consistent [14, 15]. Consequently, if a condition is valued more negatively by the general population, more favorable conclusions can be expected regarding the efficacy of an intervention for a somatic condition when using the valuation of the population rather than the patient perspective.

Much less is known about valuations of depression [16] and the role of individuals’ own state of depression in making such valuations. Two relevant studies have investigated depression valuations, both showing that individuals who experience depression value hypothetical depression states more negatively that those who do not [17, 18]. This less negative valuation of depression by the general population entails that evaluations of depression interventions, in contrast to interventions for somatic conditions, would result in conclusions of less efficacy when using the population’s perspective. However, current evidence is insufficient to draw firm conclusions, given the limited number of studies and their methodological shortcomings. In the previous studies, discrepancies were observed between individuals without depression and individuals experiencing severe levels of depression. Furthermore, a limited number of hypothetical depression states were included (mild, moderate and severe depression), which may not have reflected the variety of different states of depression, characterized by different levels of dysfunction across health-related quality of life domains. Moreover, discrepancies were more prominent when a rating scale was used rather than the standard gamble method, although the latter method has been considered more appropriate [19]. Thus, the first aim of this study was to investigate whether discrepancies in valuations of depression occur between individuals with and without depression.

The existence of discrepancies in valuations made by individuals with and without depression, if found, may carry policy implications, making the question of whose values to use in evaluations more critical. To inform this choice of perspective, it is also important to understand the underlying mechanisms of the discrepancies, which was the second aim of this study. Relevant research, although limited, exists regarding valuations for somatic conditions, in which a number of factors have been suggested as influencing over- and/or under-valuations by the patients or by the general population [20, 21]. Patients’ adaptation to their conditions [22] as well as the general population’s attention to the negative aspects of the conditions [23] have both been found to explain why patients value somatic conditions less negatively than the population. For depression, some suggestions have thus far been made concerning factors that account for observed discrepancies in valuations between individuals with and without depression. The effect of these suggested factors has never been empirically investigated. Among such factors, dysfunctional attitudes (for example, black and white thinking) have been suggested to relate to downward bias in valuations of individuals with depression, whereas stigma toward depression (for example: “They could snap out of depression if they really wanted”) has been proposed to account for upward biases in valuations of individuals without depression [18].

Following and expanding on this line of thought, we hypothesized that potential discrepancies in valuations of depression can be explained by differences between individuals with and without depression with regard to how they perceive depression and with regard to their individual characteristics. We expected that depression, like other mental conditions with an unclear nature and cause, can be more prone to subjectivity in how it is perceived by those with no experience of it. More specifically, we expected that individuals without depression might differ from those with depression with regard to stigma (for example, whether depression is a “real” disease), empathy (the degree to which the experience of being in depression can be understood), perceived susceptibility (whether depression can occur to oneself) and illness perceptions (whether depression is treatable, what its consequences are). We, therefore, hypothesized that this subjectivity in the perceptions of depression by individuals who do not experience it could be related to the discrepancies in valuations of depression between individuals with and without depression. In addition, we expected that individuals who experience depression would differ from those who do not experience depression with regard to individual characteristics known to relate to depression, specifically levels of mastery, self-compassion and dysfunctional attitudes. We expected lower levels of mastery and self-compassion and higher level of dysfunctional attitudes among individuals with depression compared with individuals without depression, and we hypothesized that these differences in characteristics might be related with the discrepancies in how they value depression. Our purpose was to determine whether the valuations of individuals with depression or the general population were affected by subjectivity in perceptions of depression, by individual characteristics related to depression or by both. We considered this question important for determining whose valuations can be considered more valid and legitimate.

Through the current study, we aimed to investigate whether individuals with and without depression differ in their valuations of depression. To overcome the limitations of previous studies on this subject, we conducted a large-scale valuation study using a wide range of hypothetical depression states based on a standard depression-specific classification system, and we elicited valuations using the time-trade-off (TTO) method. Furthermore, we examined whether potential differences can be related to group differences in perceptions of depression, by individual characteristics or by both.

## Methods

### Participants and procedures

This project consisted of a cross-sectional nationwide study conducted among members of the Dutch population. Participants included in this study were at least 18 years old, were able to understand Dutch and provided informed consent. Recruitment was conducted via an existing panel of an international company specialized in sampling for marketing and academic research [24]. To ensure representativeness of the sample, we used a stratified sampling strategy with strata based on gender, age, educational background and place of residence (across the 12 provinces of the Netherlands). In the first part of the survey, we collected data on participants’ demographic characteristics and health statuses. In the second part, after being trained in the valuations tasks, each participant valued a total of four vignettes describing different states of depression. In the third part, participants filled out a questionnaire related to their experiences with chronic conditions and depression, their perceptions of depression and their individual characteristics. The study protocol was reviewed by the Medical Ethical Committee of the University Medical Center Groningen, and a waiver was provided (M12.119685). We pilot-tested the instruments with 200 participants. Data regarding the difficulty of the tasks, completion time and the construct validity of valuations supported the feasibility of the study protocol. These data were only used for pilot purposes. Primary data collection took place online using software developed for this study by the same company that recruited the participants.

### Vignettes of depression health states

Thirty vignettes with descriptions of different depression states were developed. Each participant was randomly presented four of a total of 30 vignettes, and the order of presentation was randomized. Depression states were based on the McSad depression-specific classification system [25], previously translated and validated in Dutch [26]. The McSad consists of six depression-specific health-related quality of life domains: *Emotion*, *Self*-*appraisal*, *Cognition*, *Physiology*, *Behavior* and *Role function*. Each domain could be categorized using one of the four levels of dysfunction, namely “no,” “mild,” “moderate” or “severe” dysfunction. Different combinations of the four levels across the six domains make it possible to generate 4096 (4^6^) different states. For example, the state with the profile “*343433*” describes moderate dysfunction in the *Emotion*, *Cognition*, *Behavior* and *Role functioning* domains and severe dysfunctioning in the *Self*-*appraisal* and *Physiology* domains. To include depression states with different combinations of the four levels of dysfunction across the six domains, we used the orthogonal procedure (SPSS), which generated 25 states based on the McSad classification system. Another five states, more consistent with respect to level of dysfunction across the six domains, were added. Table 1 provides an example of the description of a depression state (“222222”).

**Table 1:** Example of two vignettes describing McSad depression state “222222” and “444444”

Emotion	You feel more down than usual and you don’t enjoy things as usual
Self-appraisal	You don’t feel very good about yourself these days and you often see the down side of everything
Cognition	You have some trouble concentrating and remembering these days, and it seems harder to make decisions
Physiology	Your sleep is a little troublesome these days. You don’t have quite the normal get up and go and you gave less of an appetite
Behavior	Things are more of a chore these days and at times you feels sluggish or agitated
Role function	You are able to function okay at work, home, school or with friends, but often don’t enjoy what you are doing
Emotion	You feel terribly down or sad all the time. You don’t enjoy anything and you feel desperate
Self-appraisal	You feel worthless and see absolutely no hope for yourself, or you don’t know why people even care about you, or you feel very guilty about the past and see no future for yourself
Cognition	You feel like your mind is shut down, overloaded or racing. You can’t read or watch TV and you can’t make even little decisions
Physiology	Your sleep is terrible these days and you don’t feel rested. You have absolutely no energy and you feel constantly tired. You have no interest in food and you have lost a lot of weight over the last month
Behavior	You can’t do anything. You are completely shut down, or extremely agitated, or, you don’t see any perspective, and wonder whether you wouldn’t be better off dead
Role function	You had to stop work, or you do nothing at home, or have completely withdrawn from everything

The 30 depression states were rated in terms of their severity (as mild, moderate or severe) by three mental healthcare professionals (two clinical psychologists and one psychiatrist), all specialized and experienced in working with individuals with depression. They first independently rated the 30 states, and inter-rater agreement was strong (Kendall’s *W* = .773) [27]. Initial discrepancies among the ratings were resolved through discussion, achieving a final consensus of four states rated as “mild,” 17 as “moderate” and nine as “severe.”

### Valuation elicitation procedure

To elicit valuations of depression, we used the TTO method, which has been shown to be a reliable and valid method [19] in previous evaluations of mental health conditions [28, 29]. In the TTO, participants are asked to imagine and choose between two options. The first option involves living in the health state described for another 10 years and then dying. The second option involves full recovery from this state, but living for less than 10 years. The number of years in the second option varies, until the point of indifference between Choice A and Choice B is reached.

In the current study, participants were trained in the use of the TTO method using a vignette describing asthma. A ping-pong iteration method was used [30]. An interactive horizontal scale representing the life years (0–10) was used as a visual aid. In the primary valuation tasks, the ping-pong method was skipped, and participants could use the interactive scale directly and report the point of indifference between Choice A and Choice B. We chose this option because the pilot testing indicated that using the ping-pong method for multiple states was tiring for participants. The value attached to the health state was calculated based on the TTO as 1 − (*x*_max_/10), where *x*_max_ is the maximum years the participant was willing to trade to live free of depression. The value could therefore range from 0 to 1, with lower values representing more negative valuations.

Upon completion, participants were asked to use a five-point scale (with 1 being “not difficult at all” and 5 being “very difficult”) to indicate difficulty with understanding the task.

### Measures

#### Defining depression groups

To assign participants to one of the two depression groups (“with depression” or “without depression”), we used the cutoff of 10 on the *Patient Health Questionnaire*—*9-item* scale (PHQ-9) [31]. The PHQ-9 is an established scale, previously validated in the Dutch language [32], consisting of nine items corresponding to DSM depression symptoms, such as reduced interest or difficulty sleeping. The questions relate to the frequency of depressive symptoms experienced during the last 2 weeks, and answers are provided on a four-point scale, ranging from 0 (“not at all”) to 3 (“almost every day”). Item scores are summed up to calculate a total score, which ranges from 0 to 27, with higher scores representing higher levels of depression. The PHQ-9 has established psychometric properties among the general population [31]. The cutoff of 10 has been recommended to indicate the presence of major depression [31, 33]. Results on the PHQ-9 in the current study demonstrated good internal consistency (Cronbach’s alpha: .92), comparable to what has been previously reported [31, 34].

#### Explanatory factors of potential discrepancies between individuals with and without depression

Empathy was assessed using the *Emotional* subscale of Batson and colleagues’ *Empathy* scale [35].The six items of the scale are words describing emotions (e.g., “sympathy”), and participants are asked to what degree they experienced each emotion (from 1: “not at all” to 6: “very much”) when reading a vignette describing a woman who experiences depression. Total scores range from 6 to 36, with higher scores representing higher levels of empathy. The *Perceived Susceptibility* scale [36] was used to assess to what extent participants perceived themselves susceptible to depression. The scale includes four items (e.g., “What do you believe is the chance that you will develop depression in your lifetime?”) with varying answer categories. Total scores range from 4 to 22, with higher scores indicating higher perceived susceptibility to depression. Cognitive representations of depression were assessed using the subscales of *Consequences*, *Treatment Control* and *Timeline Cyclical* from the revised *Illness Perception Questionnaire* (IPQr) [37], Dutch version [38]. The number of items differs per subscale (example item: “Depression does not have much effect on one’s life”). Total scores for the *Consequences*, *Treatment Control* and *Timeline Cyclical* subscales range from 6 to 30, 5 to 25 and 4 to 20, respectively. The *Stigma for Depression* scale [39], used to assess personal stigma for depression, includes nine items (e.g., “People with depression could snap out of it if they wanted”), with a tenth item added by our group following content validity assessments. The scale’s total score ranges from 0 to 40, with higher scores indicating higher stigma. Mastery was measured by means of the Dutch version [40] of the *Pearlin Mastery* scale [41]. The scale includes seven items (e.g., “I have little control over things that happen to me”), with five answer categories and total scores ranging from 7 to 35. Higher scores indicate higher mastery. The *Brief Self-Compassion* scale, available in Dutch [42], was used to assess self-compassion. It includes 12 items, to be answered on a seven-point scale. The total score is computed as the mean of item scores and, therefore, ranges from 1 to 7, with higher scores indicating higher self-compassion. Dysfunctional attitudes were assessed using the *Perfectionism* subscale of the Dutch version of the *Revised Dysfunctional Attitudes* scale [43]. The scale consists of eleven items (e.g., “If I fail at my work, then I am a failure as a person”), and answer categories range from 1 to 7. Total scores ranges from 11 to 77, with higher scores indicating less dysfunctional attitudes. Validated Dutch versions were used for all of the scales. As validated Dutch versions were not available for the scales assessing *Stigma for Depression*, *Perceived susceptibility* and *Empathy,* we used established translation guidelines [44, 45] to forward–backward translate these scales into Dutch and then back into English.

#### Additional information

Information was collected on participants’ gender, age, nationality, place of residence, education, family situation, occupation and religious background. Generic health status was also assessed by means of the EQ-5D + C variant health status classification system and visual analogue scale [46, 47]. Additional questions concerned previous diagnoses of a list of 23 chronic conditions, other than depression. Furthermore, we collected self-reported information concerning previous diagnoses of depression by a psychiatrist or a psychologist, previous depressive episodes or experience with depression via close friends or family. This information was collected to better describe our sample and to control for their effect when searching for discrepancies in valuations based on depression status.

### Analysis plan

To answer our first research question concerning discrepancies in valuations of depression between individuals with and without depression, we first defined the two depression groups, described them and compared them to each other and to the general population, using Chi-square and *t* tests. We then aimed to test the effect of the depression group (defined as the predictor variable) on valuations (defined as the outcome variable). Given that the valuations represent a scale from 0 to 1 using discrete increments of .05, the frequency distribution of the valuations was approximated with a binomial distribution in which the proportion represents a 0–1 scale. A binomial distribution would approximate a normal distribution when no floor or ceiling effects are present. The binomial proportions were modeled using a logit link function. To account for the correlation among the four valuations per subject, a random intercept for subjects was included in the binomial proportion. The effect of the depression group on valuations, including an interaction term with the severity of the depression state, was tested using the Wald test, controlled for demographic variables that were related to both the depression group and the valuations, as well as for the depression states within the severity groups. The mean valuations were estimated for the “with depression” and the “without depression” groups separately for mild, moderate and severe states of depression. To reduce the possibility of biases in valuations due to inappropriate completion of the TTO task, we repeated the analyses, excluding those participants who were not willing to trade any years or who traded the same number of years for all four of the vignettes presented to them.

When the effect of the depression group was significant, for all severity states or just one severity group, we repeated the same analysis for the relevant severity groups, by examining the effect of variables related to perceptions of depression and individual characteristics on the valuations. Both the main effect of these variables and their interaction with the depression group were investigated. We used the basic model previously described, with valuations as the outcome variable and demographic variables and depression group as predictor variables. To this model, we added each of the six variables (empathy, perceived susceptibility, illness perceptions, mastery, self-compassion and dysfunctional attitudes) one by one as predictors, to examine for main effects. To examine interactions, for each of these six variables, we added an interaction term with depression group to our model. Our hypothesis was that certain variables would affect the valuations of either the “with depression” or the “without depression” groups, resulting in discrepancies in valuations between the two groups. For example, dysfunctional attitudes might affect valuations of individuals with depression, resulting in lower valuations. Therefore, we examined the depression group (“with depression” or “without depression”) as a moderator in the relationship between the aforementioned variables and valuations of depression. For this purpose, we examined the interaction effect of each of the aforementioned variables, with the depression group as a predictor of depression valuations.

The analysis was conducted using the GLIMMIX procedure of the SAS/STAT^®^ software [48], a generalized linear mixed model that extends the class of generalized linear models (GLMs) by incorporating normally distributed random effects. This procedure was considered appropriate, given the structure of our data, specifically the correlations among the responses. A difference of .05 in valuations was considered meaningful [49, 50], and statistical significance was assessed at the .05 level.

## Results

### Sample

**Fig. 1:**
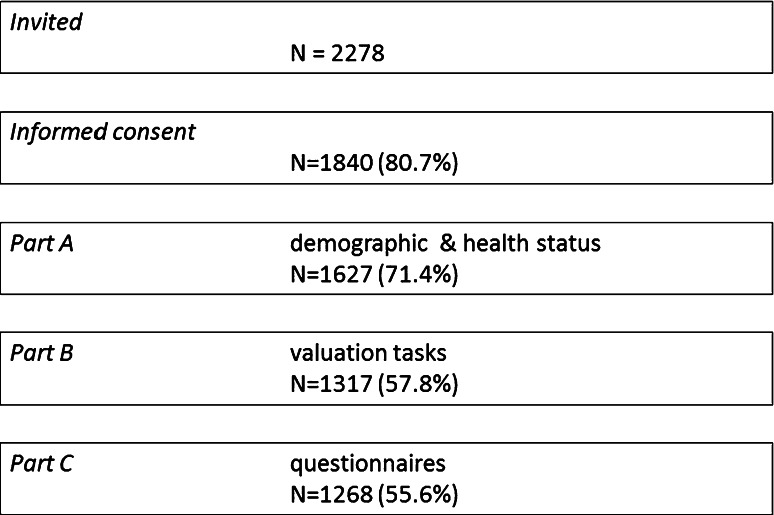
Study flow diagram

From the 2278 individuals who were invited to participate, 1268 (55.6 %) completed the survey. A flowchart of dropouts for the different stages of the survey is presented in Fig. 1. Table 2 provides a description of the final sample. Chi-square tests confirmed the representativeness of the sample within the Dutch population with regard to age, gender, place of residence and educational background. Using the PHQ-9 cutoff score of 10, 200 participants (15.8 %) were assigned to the “with depression” group, a percentage relatively higher from the 10 % previously reported [34]. In the whole sample, the majority of participants reported either no (44.2 %) or minor (24.5 %) difficulties with understanding the valuation task.

**Table 2:** Description of the two depression groups

	With depression		Without depression		Compare^a^	Sample (%)	Population (%)	Compare^b^
	*N* = 200	%	*N* = 1068	%				
General								
Gender								
Female	119	59.5	529	49.5	*χ* ^2^(1) = 6.70 *p* = .011	51.1	51	*p* = .76
Age								
Mean (SD)	42.04 (16.17)		47.53 (17.50)		*t* = 4.35 *p* < .000			
18–35	71	35.5	287	26.9	*χ* ^2^(3) = 26.79 *p* < .000	28.2	27.0	*p* = .98
36–50	75	37.5	296	27.7		29.3	28.0	
51–65	38	19.0	264	24.7		23.8	25.0	
65+	16	8.0	221	20.7		18.7	20.0	
Education								
Low	76	38.0	340	31.8	*χ* ^2^(2) = 5.67 *p* = .059	32.8	34.3	*p* = .95
Middle	86	43.0	446	41.8		42.0	42.1	
High	38	19.0	282	26.4		25.2	23.5	
Family situation								
With partner	109	54.5	704	65.9	*χ* ^2^(1) = 9.54 *p* = .002			
Single/other	91	45.5	364	34.1				
Children								
Yes	95	54.7	650	60.9	*χ* ^2^(1) = 12.41 *p* = .001			
Occupation								
Paid job	50	25.0	413	23.7	*χ* ^2^(2) = 13.62 *p* = .001			
Education	26	13.0	118	13.7				
Other	124	57.0	537	62.6				
Religion								
Yes	63	31.5	443	41.5	*χ* ^2^(1) = 7.00 *p* = .009			
General health								
Chronic conditions								
Yes	141	70.5	546	51.1	*χ* ^2^(1) = 25.48 *p* < .000			
EQ-6D VAS								
Mean (SD)	63.17 (18.53)		80.99 (15.22)		*t* = 12.813 *p* = .000			
Depression								
Under treatment	72	36.0	59	8.1	*χ* ^2^(1) = 101.5 *p* < .000			
Diagnosed-ever	108	54.0	179	16.8	*χ* ^2^(1) = 133.4 *p* = .000			
Episode-ever	152	76.0	370	34.6	*χ* ^2^(1) = 118.9 *p* < .000			
Experience-others	113	56.5	323	30.1	*χ* ^2^(1) = 51.47 *p* < .000			

^a^Compare “with depression” and “without depression” groups

^b^Compare sample (whole) to the general Dutch population

### Discrepancies in valuations between individuals with and without depression

Among the participant variables, age, family situation and experience with chronic conditions were found to be correlated with depression group (“with depression” or “without depression”) and with valuations. We, therefore, controlled for these three variables in our analysis. Additionally, we controlled for the different depression states within the same severity group. Results of the generalized linear mixed models (GLMM) analysis demonstrated a significant interaction effect between the severity of the depression states and the depression group on valuations of depression (*F* = 3.22; *p* = .04). This interaction effect indicated that the effect of the presence of depression on valuations was different among depression states of different severity. A significant and clinically meaningful difference in estimated mean values between “with depression” and “without depression” groups was observed for mild, but not for moderate or severe depression states (see Table 3). Individuals with depression valued mild depression lower, and thus more negatively, compared with individuals without depression. When we repeated the same analysis excluding participants who were not willing to trade any years (*N* = 104, 8.2 %) or who traded the same number of years for all four of the states presented to them (*N* = 154, 12.1 %), only minor changes in the results were observed. The interaction effect between the severity of depression states and the depression group on valuations of depression remained significant, and similar differences were found in estimated mean values between the two depression groups.

**Table 3:** Estimated mean valuations^a^ for the “with depression” and the “without depression” group, separately for groups of depression states of different severity

Severity of the depression state	With depression	Without depression	*p* value
	M (SD)	M (SD)	
Mild	.693	.776	.007
Moderate	.653	.709	.080
Severe	.604	.659	.111

^a^Based on the GLMM; controlled for age, family situation, experience with chronic conditions and the states within severity groups

### Explanatory factors for discrepancies between individuals with and without depression

Depression groups differed in their average scores on all of the variables related to perceptions of depression and individual characteristics, except for stigma and perceptions concerning treatment control (see Table 3). As hypothesized, individuals without depression reported lower levels of empathy and perceived susceptibility, and they perceived depression to be related to fewer consequences and a more cyclical timeline. Individuals with depression showed lower levels of mastery and self-compassion and higher levels of dysfunctional attitudes.

After controlling for age, family situation, experience with chronic conditions and the depression states within the severity groups, we examined the variables related to perceptions of depression and individual characteristics as predictors of depression valuation, separately for each level of depression (mild, moderate and severe). Self-compassion (*p* = .045) and dysfunctional attitudes (*p* = .013) were found to significantly affect valuations of mild depression, with higher levels of self-compassion and less dysfunctional attitudes related to higher, and thus less negative, valuations. Perceptions concerning consequences of depression (*p* = .020) and treatment control (*p* = .001) were found to affect valuations of moderate depression states, with higher valuations related to stronger beliefs that depression has major consequences and that treatment can control depression. Empathy (*p* = .022) and perceived susceptibility (*p* = .002) were found to affect valuations of severe depression states, with higher valuations related to less stigma for depression and lower levels of perceived susceptibility to depression. When examined, the interaction term for each of these predictor variables with the depression group was never found to be significant, meaning that for none of these variables was their association with valuations different for the two groups. This result held for all of the different levels of depression severity. Therefore, the depression groups was not found to moderate the effect of the predictor variables on valuations, or in other words, the effect of the predictor variables on depression valuations was not different between individuals with and without depression. This implies that discrepancies in valuations did not seem to relate to differences between individuals with and without depression with regard to their perceptions of depression or to their individual characteristics.

## Discussion

Results of this study answer our first research question concerning discrepancies between individuals with and without depression in valuations of depression states. Specifically, results show that individuals with and without depression differ in their valuations of mild hypothetical depression states, with individuals with depression valuing mild states of depression more negatively than those without depression. For moderate and severe depression states, no discrepancies between individuals with and without depression were observed. Concerning the second research question, results show that, although individuals with and without depression differ in their perceptions of depression and their individual characteristics, these differences do not account for the observed discrepancies in their valuations. Our results are in line with and add specificity to results from previous studies showing more negative valuations of hypothetical depression states by individuals who experience depression. Previous studies report that discrepancies are dependent on the severity of the hypothetical depression state, with discrepancies mainly observed for hypothetical states of mild severity. In addition, discrepancies were previously reported only between individuals without depression and a specific subgroup of individuals with depression, rather than between the two groups [17, 18]. In this study, we compared individuals with and without depression overall, rather than subgroups, and we found that discrepancies do exist for valuations of mild depression. This finding is more valuable than those from previous studies, as the research question seeks to compare and to identify whether discrepancies occur between those who experience depression and those who do not, to understand the implications of choosing one of the two perspectives. Additionally, we examined the effect of the severity of the valued state.

This study was the first to investigate whether observed discrepancies between individuals with and without depression in valuations of depression can be explained by factors related to either the perceptions of depression by those who do not experience it (e.g., stigma or low empathy) or to individual characteristics known to be related to depression (e.g., low mastery or higher dysfunctional attitudes). As expected, individuals with and without depression differed with respect to how they perceive depression, with individuals without depression holding lower levels of empathy, higher levels of perceived susceptibility, lower perceived consequences of depression and the perception of a less cyclical timeline. With respect to their individual characteristics, individuals without depression showed higher levels of mastery and self-compassion and lower levels of dysfunctional attitudes. However, the effect of these factors on how the individuals value depression was not different between individuals with and without depression for any of the three severity levels of the hypothetical depression states. This finding implies that, although discrepancies occur between individuals with and without depression in their perceptions of depression and their individual characteristics and discrepancies also occur in their valuations of depression, the earlier differences cannot explain the latter, as hypothesized.

One of the strengths of the current study is the inclusion a large sample of participants, representative of the Dutch population with respect to age, gender, place of residence and educational background. Furthermore, the sample included a group of participants with depression that varied with respect to the years since diagnosis and the state of treatment. In contrast to previous studies, we have included a large number of depression states, ensuring a large variance with respect to the level of dysfunction in the various domains, thus supporting the generalizability of our findings.

When interpreting the results of the current study, certain limitations should be considered. First, although we used the recommended cutoff score of an established scale to assess depression [51], inclusion of participants diagnosed with depression by a healthcare professional might have been more accurate. In addition, our results might have been affected by the fact that participants recognized to experience depression might not have been aware that their symptoms can constitute a diagnosis of depression. Furthermore, following the standard TTO formulation, we asked participants to imagine living in the described states of depression for another 10 years. Yet if, as in reality, depression were described as a condition with fluctuations between depression and remission, different valuations might have been obtained. The range of observed mean values between the mildest and the most severe depression states was rather small (a range of .15 for the “without depression” group and of .13 for the “with depression” group). In previous studies, the differences between the mildest and the most severe states were also relatively small. In the study by Gerhards et al. [17], the largest difference was 12.52 in a 100-point rating scale and was observed in the group with no depression, while the smallest difference was 4.98, observed in the group with severe depression. In the study by Pyne et al. [18], maximum difference in standard gamble-based valuations between the mild and severe depression state was observed in the general population group (.24), whereas minimum differences were found in the group with severe depression (.19). This finding might be explained by the condition-specific nature of the description, but might also have made it more difficult to recognize discrepancies in valuations between the two groups. Interview-based administration of the TTO task should also be discussed. Computer-based administration of the TTO is currently the standard, and based on previous studies [52–54], we expected that online administration would be valid and thus cost-effective. However, given that the TTO is undoubtedly a cognitively demanding task, the presence of an interviewer might ensure more accuracy in comprehension. Nevertheless, the pilot testing, the logical relationships of valuations with severity of depression and participants’ reports concerning the difficulty of the task are reassuring. Finally, we found no significant interaction effects of variables related to perceptions of depression and individual characteristics with depression group on valuations. This led us to conclude that the tested interaction variables cannot explain the observed discrepancies in depression valuations between individuals with and without depression. However, it cannot be ruled out that the interaction effects were not significant due to the limited power of our study.

Our findings are of major importance for at least two reasons. Given that mild depression is valued differently according to the population being asked, we can expect less favorable outcomes if valuations are obtained from the general population, rather than from individuals with depression. This effect can be more profound in the case of interventions specifically targeting mild depression [55]. In contrast, in the case of interventions targeting individuals with moderate and severe depression, the choice of population does not seem to have an impact. Nevertheless, given that the population’s perspective correlates with more negative valuations in the case of somatic conditions, choosing the population’s perspective may entail not prioritizing mental health interventions in the agenda of health policy makers. Secondly, although we have hypothesized that various variables related to perceptions of depression and individual characteristics would explain discrepancies in perception, we found no evidence for their role. One possible conclusion is that discrepancies are not caused by upward biases in the general population, related to how they perceive depression, neither by downward biases of the individuals experiencing depressions, related to their individual characteristics. Rather, the discrepancies simply reflect genuine differences in how patients and the general population value depression, which is affected by experiences with depression itself. Future studies could therefore focus on different types of factors, such as focusing illusion [23], with individuals with depression focusing on the aspects in their health-related quality of life that are the most negatively affected by depression, while individuals without depression also consider aspects not affected by the condition.

Future investigations could possibly examine discrepancies in valuations of depression between the general population and inpatients of mental health institutions, as patients’ awareness of their diagnosis as well as their perceptions of depression might differ. Future studies should also examine to what degree the discrepancies observed in the current study can actually affect conclusions regarding the effectiveness of depression interventions. More research could also elaborate on our finding that valuations of depression are affected by the individual’s experience with other chronic conditions. Because depression often co-occurs with somatic conditions [56, 57] and special psychological interventions are developed and offered to patients with somatic condition [58], further exploration of the effect of individuals’ somatic conditions on valuations of depression is warranted. Subsequent studies could use qualitative and mixed-methods approaches to identify potential determinants of group discrepancies in valuations not considered in this study. Furthermore, examination of whether our findings concerning discrepancies and their determinants also apply to other mental health conditions would be of relevance.
